# Therapeutic coma in non-convulsive status epilepticus is associated with increased complications and mortality: a retrospective cohort study

**DOI:** 10.1007/s00415-026-14090-8

**Published:** 2026-09-06

**Authors:** Eyad Altarsha, Norma J. Diel, Minja Braun, Daniela Schoene, Alia Kaadan, Haidar Moustafa, Simon Winzer, Annahita Sedghi, Timo Siepmann, Martin Lesser, Hagen B. Huttner, Kristian Barlinn

**Affiliations:** 1 Department of Neurology, Faculty of Medicine and University Hospital Carl Gustav Carus, Technische Universität Dresden, Dresden, Germany; 2 Department of Anesthesiology, St Joseph-Stift Hospital Dresden, Dresden, Germany; 3 Department of Neurology, Clinic Tharandter Wald-Hetzdorf, Halsbruecke, Germany

**Keywords:** Non-convulsive status epilepticus, Therapeutic coma, Neurocritical care, In-hospital mortality, Medical complications

## Abstract

**Introduction:**

While therapeutic coma is an established escalation strategy in generalized convulsive status epilepticus, evidence for benefit in non-convulsive status epilepticus (NCSE) is limited and treatment-related complications may affect outcome.

**Methods:**

We performed a retrospective cohort study of patients with NCSE treated at a tertiary hospital. Patients were classified according to receipt of therapeutic coma, defined as continuous intravenous and/or inhalational anesthetic administration for seizure control. The primary outcome was the occurrence of predefined clinically relevant in-hospital medical complications, including pneumonia, sepsis, cardiac arrhythmias, acute renal failure, renal replacement therapy, venous thromboembolism and cardiopulmonary resuscitation. Secondary outcomes included in-hospital mortality, ICU length of stay and successful termination of NCSE. Confounding by indication was addressed using inverse probability of treatment weighting (IPTW) based on propensity scores.

**Results:**

Among 283 patients with NCSE, 111 (39.2%) received therapeutic coma. In IPTW-adjusted analyses, therapeutic coma was associated with higher odds of pneumonia (OR 20.40, 95% CI 8.93–46.58; *p* < 0.001), sepsis (OR 6.09, 95% CI 3.14–11.80; *p* < 0.001), cardiac arrhythmias (OR 13.96, 95% CI 4.57–42.65; *p* < 0.001), acute renal failure (OR 4.17, 95% CI 1.85–9.40; *p* = 0.001), renal replacement therapy (OR 4.97, 95% CI 1.65–14.97; *p* = 0.004) and cardiopulmonary resuscitation (OR 11.22, 95% CI 2.35–53.63; *p* = 0.002), whereas the association with venous thromboembolism did not reach statistical significance (OR 3.04, 95% CI 0.97–9.51; *p* = 0.057). Therapeutic coma was also associated with increased in-hospital mortality (RR 1.86, 95% CI 1.28–2.71; p=0.001) and prolonged ICU length of stay (*β* = 1.36, 95% CI 1.13–1.60; *p* < 0.001). Successful NCSE termination occurred less frequently in patients treated with therapeutic coma (70.3% vs. 84.3%; *p* = 0.005).

**Conclusion:**

In NCSE, therapeutic coma was associated with higher complication rates, longer ICU stay and increased in-hospital mortality, while no higher rates of short-term seizure control were observed.

**Supplementary Information:**

The online version contains supplementary material available at https://doi.org/10.1007/s00415-026-14090-8.

## Introduction

Status epilepticus (SE) is a neurological emergency associated with substantial morbidity, mortality and healthcare resource utilization [1–3]. While timely escalation of treatment is critical, outcome largely depends on seizure type, underlying etiology and the intensity of therapeutic interventions [4]. In recent years, increasing availability of continuous electroencephalography has led to improved detection of non-convulsive status epilepticus (NCSE), particularly in critically ill patients, where clinical manifestations may be subtle or nonspecific, potentially leading to delayed treatment initiation [5]. In contrast to refractory generalized convulsive SE, for which escalation to therapeutic coma is an established and often life-saving intervention, its role in refractory NCSE remains highly controversial [6]. Current national and international guidelines emphasize that treatment strategies in NCSE should be individualized and etiology driven, explicitly noting that anesthetic therapy may not always be required [7]. Nevertheless, therapeutic coma is frequently applied in clinical practice, particularly in refractory cases, despite limited evidence supporting its benefit in this population.

While aggressive seizure suppression may theoretically reduce ongoing neuronal injury, therapeutic coma is associated with well-recognized risks, including prolonged mechanical ventilation, infectious complications, cardiovascular instability and multiorgan dysfunction [8, 9]. Previous observational data, including analyses stratified by SE semiology, suggest that therapeutic coma may be associated with unfavorable outcomes, particularly in non-comatose NCSE phenotypes [10]. However, as treatment escalation is typically guided by clinical severity and refractoriness, separating treatment-related effects from underlying disease burden remains challenging [11]. Consequently, the balance between potential benefits and predictable risks of therapeutic coma in NCSE remains poorly defined.

To address this knowledge gap, we conducted a retrospective cohort study of adult patients with NCSE treated at a tertiary university hospital over a 10-year period. The primary objective was to evaluate the association between therapeutic coma and clinically relevant outcomes, including medical complications, intensive care unit length of stay and in-hospital mortality. To mitigate confounding by indication, we additionally applied propensity score-based methods to adjust for baseline differences between patients treated with and without therapeutic coma.

## Methods

### Patients and study design

We conducted a retrospective observational cohort study of adult patients (≥ 18 years) treated for NCSE at a tertiary referral center with dedicated neurocritical care and epilepsy monitoring units. Patients treated between January 1, 2010, and March 31, 2020, were identified through a systematic search of the hospital information system using ICD-10 codes G41.0, G41.1, G41.2, G41.8 and G41.9. Demographic, clinical and procedural variables were extracted from electronic medical records. The study cohort comprised consecutive NCSE admissions during the study period; the comparison group consisted of NCSE patients managed without initiation of therapeutic coma within the same cohort and time frame. This study was conducted and reported in accordance with the Strengthening the Reporting of Observational Studies in Epidemiology (STROBE) guidelines.

### Diagnosis of non-convulsive status epilepticus

The diagnosis of NCSE was based on contemporaneous electroclinical assessment integrating clinical presentation, EEG findings and treatment response as documented in the medical record. The study was conducted at a tertiary academic neuroscience center with dedicated epilepsy monitoring facilities and continuous EEG capabilities. EEG recordings were routinely interpreted by board-certified neurologists with expertise in electroencephalography and neurocritical care, including after-hours review through an on-call EEG service. NCSE was defined as a persistent alteration of consciousness, behavior or cognition associated with electrographic evidence of ongoing seizure activity in the absence of prominent motor manifestations. Diagnosis was established using continuous or repeated scalp EEG recordings interpreted according to established electroclinical criteria available at the time [11, 12]. Following their publication, the Salzburg Consensus Criteria were incorporated into routine EEG interpretation and applied alongside contemporary clinical assessment [12]. Electrographic diagnosis required continuous or near-continuous epileptiform discharges, rhythmic or periodic patterns, or evolving ictal EEG activity considered consistent with ongoing seizure activity in a compatible clinical context. In cases with equivocal EEG findings, diagnosis required additional electroclinical features to support an ictal interpretation rather than isolated periodic EEG patterns alone. Continuous EEG monitoring was performed whenever clinically indicated, particularly in critically ill patients and those treated in the intensive care unit. NCSE semiology was classified as NCSE with coma or NCSE without coma according to the level of consciousness at presentation, consistent with current ILAE terminology. The onset of NCSE was defined as the time of first documented clinical alteration or EEG confirmation, whichever occurred earlier. Etiology was classified according to the five-category framework recently proposed [13], categorizing NCSE as acute, remote, progressive, defined electroclinical syndrome, or unknown, determined on the basis of clinical history, neuroimaging, laboratory findings, and EEG data. Antiseizure medications were defined as conventional oral or intravenous antiseizure drugs used for seizure control, including levetiracetam, valproate, phenytoin, lacosamide, brivaracetam, carbamazepine/oxcarbazepine, gabapentin, perampanel, topiramate and lamotrigine. Continuous anesthetic agents used for therapeutic coma were not considered part of ASM burden. Likewise, benzodiazepines administered for initial seizure termination were not included, as ASM burden was intended to quantify escalation of conventional antiseizure therapy beyond first-line emergency treatment.

### Therapeutic coma

The decision to initiate therapeutic coma was at the discretion of the treating physicians based on clinical judgment and EEG findings. Therapeutic coma was defined as continuous administration of anesthetic agents for seizure control, administered either intravenously (propofol, midazolam, barbiturates, ketamine), via inhalational anesthetics (sevoflurane or isoflurane) or as a combination of both. During the study period, anesthetic therapy was titrated according to contemporaneous institutional practice using bedside EEG monitoring, with EEG-guided burst suppression commonly pursued as the therapeutic target. Duration of therapeutic coma was recorded in hours. For exploratory dose–response analyses within the therapeutic coma group, coma exposure was additionally expressed as the number of 24-h “coma cycles” defined as total coma hours divided by 24 (i.e., each initiated 24-h period counted as one cycle).

### Study outcomes

#### Primarye outcome

The primary outcome was the occurrence of clinically relevant complications during hospitalization. Predefined complications included pneumonia, sepsis, cardiac arrhythmias requiring intervention, acute kidney injury, need for renal replacement therapy, venous thromboembolism, cardiopulmonary resuscitation (CPR), gastrointestinal complications (ileus or bowel ischemia) and acute liver failure.

More specifically, pneumonia was defined by the presence of compatible clinical signs, laboratory abnormalities and radiographic evidence. Sepsis was identified according to contemporaneous consensus definitions requiring documented infection and systemic inflammatory response with organ dysfunction. Acute kidney injury was classified according to Kidney Disease: Improving Global Outcomes (KDIGO) criteria, and renal replacement therapy was recorded when intermittent or continuous dialysis was initiated during hospitalization. Cardiac arrhythmias comprised clinically significant rhythm disturbances requiring pharmacological or electrical treatment. Cardiopulmonary resuscitation was assessed as a separate outcome reflecting cardiac arrest events during hospitalization. Venous thromboembolism was defined as confirmed deep vein thrombosis or pulmonary embolism. Gastrointestinal and hepatic complications were diagnosed based on standard clinical, laboratory and imaging criteria. All endpoints reflected diagnoses established during routine clinical care by experienced intermediate and/or neurocritical care physicians in accordance with established diagnostic standards [14–19].

#### Secondary outcomes

Secondary outcomes comprised in-hospital mortality from any cause, ICU length of stay (LOS) and successful termination of NCSE. Successful termination of NCSE was determined from the medical records based on the treating physicians' documentation and the available electroclinical information. Resolution was defined as sustained absence of ongoing NCSE without recurrence until hospital discharge. Where available, EEG findings contributed to this assessment together with the documented clinical course. Circumstances of death were extracted from physician documentation and categorized as withdrawal/limitation of life-sustaining treatment (WLST) versus death under ongoing intensive care due to medical complications.

Treatment intensity was assessed by ASM burden, defined as (i) the maximum total number of ASMs administered during hospitalization and (ii) the maximum number of concurrently administered ASMs. These measures were used to describe treatment escalation and to define a sensitivity analysis restricted to patients acutely treated with ≥ 3 ASMs as a pragmatic proxy for refractory disease.

### Statistical analysis

Continuous variables are reported as means with standard deviations or medians with interquartile ranges (IQR), as appropriate, and categorical variables as frequencies and percentages. Unadjusted comparisons between groups were performed using chi-square or Fisher’s exact test for categorical variables and Student’s t-test or Mann–Whitney U test for continuous variables. To address confounding by indication, inverse probability of treatment weighting (IPTW) based on propensity scores was applied. Propensity scores for receiving therapeutic coma were estimated using logistic regression including covariates selected a priori based on clinical relevance: age, sex, NCSE etiology, NCSE semiology (with vs. without coma), history of epilepsy and pre-admission ASM use. Stabilized inverse probability weights were calculated and trimmed at the 1 st and 99th percentiles to limit the influence of extreme weights. Covariate balance before and after weighting was assessed using standardized mean differences, with values <0.1 indicating adequate balance. An exploratory multivariable logistic regression model was additionally fitted to identify factors independently associated with initiation of therapeutic coma. Weighted regression models were then used to evaluate associations between therapeutic coma and outcomes. Binary outcomes were analyzed using weighted Poisson regression (to estimate relative risks) and weighted logistic regression (odds ratios) with robust variance estimation. ICU LOS was analyzed using weighted linear regression of log-transformed ICU LOS (log[ICU LOS + 1]). Sensitivity analyses were conducted in three separate analyses: restriction to intubated patients (A), restriction to patients treated with ≥3 ASMs (B), and exclusion of patients with hypoxic NCSE (C). For each sensitivity analysis, propensity scores were re-estimated within the respective restricted cohort, stabilized inverse probability weights were recalculated, and all IPTW-adjusted outcome models were refitted using the newly derived weights. Exploratory analyses within the therapeutic coma group assessed dose–response associations between therapeutic coma duration, modeled both continuously (hours) and categorically (24-h cycles) and selected medical complications. All analyses were conducted using available-case analysis. No imputation was performed. Propensity score estimation and IPTW outcome models included patients with non-missing values for the respective covariates and outcomes. Circumstances of death were analyzed among decedents with available cause-of-death categorization. All tests were two-sided and a *p* value <0.05 was considered statistically significant. Analyses were performed using Stata SE version 16.0 (StataCorp LLC, College Station, TX, USA).

## Results

### Study population

**Fig. 1:**
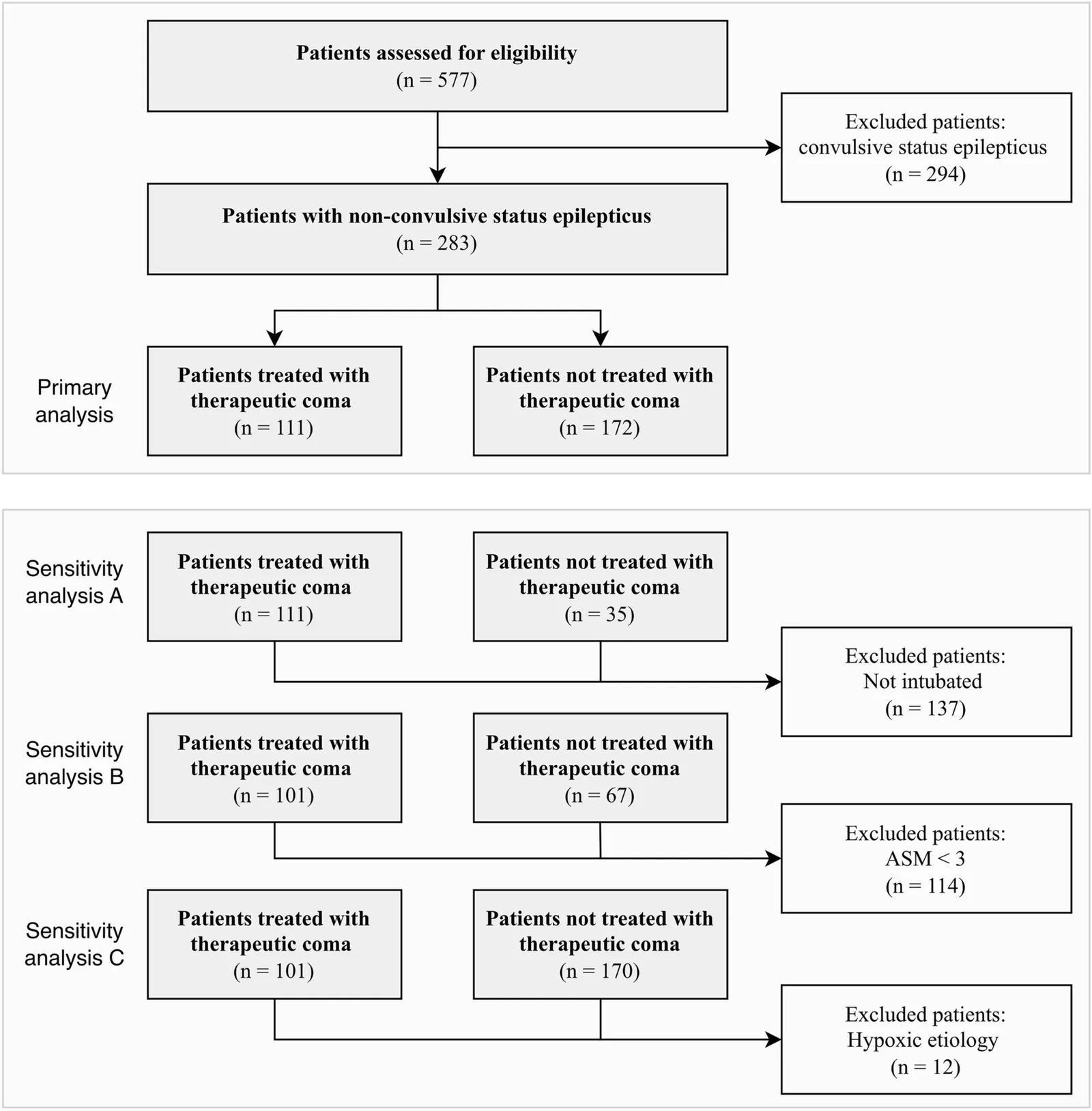
Flowchart illustrating patient selection, exclusions and allocation to therapeutic coma and non-coma treatment groups

A total of 283 adult patients with NCSE were included in the analysis, of whom 111 (39.2%) were treated with therapeutic coma and 172 (60.8%) were managed without therapeutic coma (Fig. 1). Baseline characteristics of the overall cohort and by treatment group are shown in Table 1. Before weighting, patients receiving therapeutic coma were younger, more frequently male, less likely to receive pre-admission antiseizure medication, more frequently presented with NCSE with coma and differed with respect to NCSE etiology. After application of stabilized IPTW, all prespecified baseline covariates were well balanced between groups, with standardized mean differences below 0.1. Standardized mean differences before and after weighting for the primary IPTW analysis are provided as Supplementary Table S1. In an exploratory multivariable logistic regression analysis, younger age and NCSE with coma were independently associated with initiation of therapeutic coma (Table 2).

**Table 1:** Baseline characteristics

	Therapeutic coma *n* = 111	No therapeutic coma *n* = 172	*p*-value
*Demographics*			
Age, years, median (IQR)	69 (58–79)	76 (68–81)	< 0.001
Male sex, *n* (%)	57 (51.4)	66 (38.4)	0.03
*Comorbidities, n (%)*			
Diabetes mellitus	33 (29.7)	60 (34.9)	0.37
Atrial fibrillation	32 (28.8)	43 (25)	0.48
Arterial hypertension	87 (77.4)	132 (76.4)	0.75
Coronary artery disease	24 (21.6)	32 (18.6)	0.53
Congestive heart failure	40 (36)	44 (25.6)	0.06
Chronic renal failure	29 (23.7)	38 (22.1)	0.44
Chronic liver disease	13 (11.7)	14 (8.1)	0.32
Current alcohol use	18 (16.2)	18 (10.5)	0.16
Current tobacco use	14 (12.6)	10 (5.8)	0.045
*Clinical presentation, n (%)*			
Admission GCS, median (IQR)	6 (3–8)	9 (5–12)	<0.001
NCSE with coma, *n* (%)	77 (69.4)	71 (41.3)	< 0.001
Prehospital intubation, *n* (%)	25 (22.5)	13 (7.6)	<0.001
*Epilepsy-related history*			
History of epilepsy, *n* (%)	33 (29.7)	65 (37.8)	0.16
Pre-admission ASM use, *n* (%)	28 (25.2)	64 (37.2)	0.036
*NCSE etiology, n (%)*			< 0.001
Acute	46 (41.4)	49 (28.5)	
Remote	42 (37.8)	74 (43.0)	
Progressive	4 (3.6)	19 (11.0)	
Defined electroclinical syndrome	3 (2.7)	7 (4.1)	
Unknown	16 (14.4)	23 (13.4)	

Values are presented as median (interquartile range) or number (percentage), as appropriate. *P*-values refer to unadjusted comparisons between groups

*ASM* indicates antiseizure medication, *NCSE* non-convulsive status epilepticus

**Table 2:** Factors associated with initiation of therapeutic coma

Predictor	OR (95% CI)	*p*-value
Age, per year	0.96 (0.94–0.98)	< 0.001
*Sex, reference: male sex*		
Female sex	0.81 (0.47–1.38)	0.49
*Etiology, reference: acute*		
Remote	0.83 (0.45–1.56)	0.57
Progressive	0.35 (0.10–1.17)	0.09
Defined electroclinical syndrome	0.28 (0.05–1.59)	0.15
Unknown	0.95 (0.42–2.14)	0.9
NCSE with coma, reference: NCSE without coma	2.76 (1.60–4.76)	<0.001
Positive history of epilepsy, reference: no history of epilepsy	2.43 (0.43–13.59)	0.31
Prior ASM intake, reference: no ASM intake	0.29 (0.05–1.62)	0.16

Odds ratios (*OR*) with 95% confidence intervals (*CI*) are derived from a logistic regression model including age, sex, non-convulsive status epilepticus (*NCSE*) etiology, history of epilepsy and pre-admission antiseizure medication (*ASM*) use

### Medical complications

**Fig. 2:**
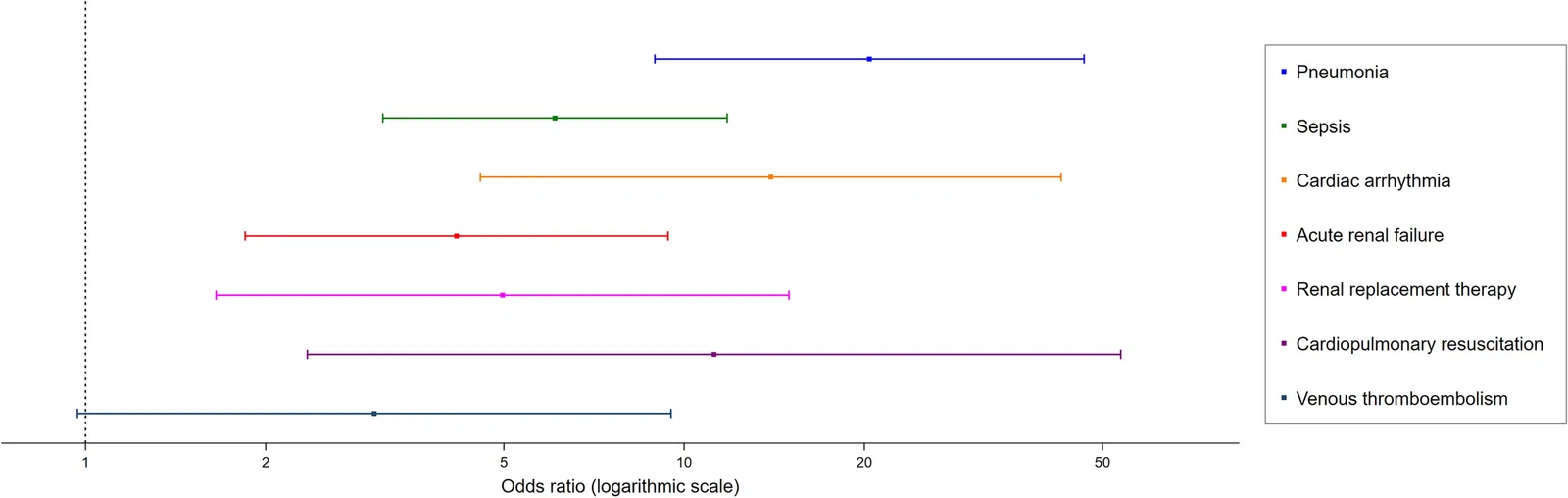
Forest plot showing odds ratios (*ORs*) with 95% confidence intervals (*CIs*) for the association between therapeutic coma and in-hospital medical complications. Estimates were derived from inverse probability of treatment weighted logistic regression models with robust variance estimation. Propensity scores were calculated using age, sex, NCSE etiology, history of epilepsy and pre-admission antiseizure medication use. The vertical red line indicates the null value (OR = 1). All models included available cases only

Medical complications occurred more frequently among patients treated with therapeutic coma. In IPTW-weighted analyses, therapeutic coma was associated with higher odds of pneumonia (OR 20.40, 95% CI 8.93–46.58; *p* < 0.001), sepsis (OR 6.09, 95% CI 3.14–11.80; *p* < 0.001), cardiac arrhythmias (OR 13.96, 95% CI 4.57–42.65; *p* < 0.001), acute renal failure (OR 4.17, 95% CI 1.85–9.40; *p* = 0.001), need for renal replacement therapy (OR 4.97, 95% CI 1.65–14.97; *p* = 0.004), venous thromboembolism (OR 3.04, 95% CI 0.97–9.51; *p* = 0.057) and CPR (OR 11.22, 95% CI 2.35–53.63; *p* = 0.002). These associations are summarized in Fig. 2. Rare complications such as ileus, bowel ischemia and acute liver failure were observed predominantly among patients receiving therapeutic coma, but were not modeled because of low event counts. Results were essentially unchanged when admission Glasgow Coma Scale was used instead of NCSE semiology in the propensity score model.

Among patients treated with therapeutic coma, longer coma exposure was associated with higher odds of pneumonia (OR 1.023 per additional hour of therapeutic coma, 95% CI 1.003–1.043; *p* = 0.021). Expressed per 24-h coma cycle, pneumonia risk increased by approximately 89% per additional cycle (OR 1.89, 95% CI 1.11–3.22; *p* = 0.018). For sepsis, the association with coma exposure showed a similar direction but did not reach statistical significance when modeled per additional hour of therapeutic coma (OR 1.009 per hour, 95% CI 0.998–1.020; *p* = 0.10). When coma duration was categorized into 24-h cycles, longer exposure was also associated with higher odds of sepsis (OR 1.46 per additional cycle, 95% CI 1.04–2.04; *p* = 0.030).

### Treatment intensity and seizure termination

Patients treated with therapeutic coma showed substantially higher treatment intensity (Table 3). Escalation to ≥ 3 ASMs occurred in 91.9% of patients treated with therapeutic coma compared with 39.0% in the non-coma group (p < 0.001). Successful termination of NCSE before hospital discharge occurred less frequently in patients treated with therapeutic coma than in those managed without coma (70.3% vs. 84.3%; *p* = 0.005). In IPTW-weighted logistic regression, therapeutic coma was associated with a lower likelihood of successful NCSE termination (OR 0.43, 95% CI 0.23–0.81; *p* = 0.008).

**Table 3:** Disease severity and treatment intensity

	Therapeutic coma *n* = 111	No therapeutic coma *n* = 172	*p*-value
*Disease severity*			
ICU admission, *n* (%)	111 (100)	116 (67.4)	< 0.001
Mechanical ventilation, *n* (%)	111 (100)	35 (20.3)	< 0.001
Median hospital LOS in days (IQR)	26 (19–40)	18 (9–28)	< 0.001
Median ICU LOS^†^ in days (IQR)	26 (19–40)	22 (13–31)	< 0.001
Median duration of SE in hours (IQR)	172.1 (113.9–327.8)	65.5 (19.1–140.6)	< 0.001
*Treatment intensity and refractoriness*			
Benzodiazepines for initial seizure termination, *n* (%)	73 (65.8)	106 (61.6)	0.48
Maximum ASMs ≥ 3*, *n* (%)	102 (91.9)	67 (39)	< 0.001
*Maximum number of ASMs during hospitalization, n (%)*			< 0.001
1–2	9 (8.1)	103 (59.9)	
3	34 (30.6)	44 (25.6)	
4	36 (32.4)	17 (9.9)	
≥ 5	32 (28.8)	6 (3.5)	
Median number of ASMs during hospitalization (IQR)	4 (3–5)	2 (1–3)	< 0.001
*Maximum number of concurrent ASMs, n (%)*			< 0.001
1–2	17 (15.3)	114 (66.3)	
3	47 (42.3)	45 (26.2)	
4	47 (42.3)	11 (6.4)	
≥ 5	3 (3–4)	2 (1–3)	
Median number of concurrent ASMs (IQR)	3 (3–4)	2 (1–3)	< 0.001
*Number of ASMs administered before therapeutic coma initiation, n (%)*			< 0.001
1–2	59 (53.2)		
3	28 (25.2)		
4	18 (16.2)		
≥ 5	6 (5.4)		
Median number of ASMs before therapeutic coma initiation (IQR)	2 (2–3)		
*ASM used (any time during hospitalization), n (%)*			
Levetiracetam	100 (90.1)	144 (83.7)	
Valproate	58 (52.3)	65 (37.8)	
Phenytoin	96 (86.5)	73 (42.4)	
Lacosamide	72 (64.9)	42 (24.4)	
Brivaracetam	12 (10.8)	3 (1.7)	
Carbamazepine/oxcarbazepine	15 (13.5)	12 (7)	
Gabapentin	9 (8.1)	2 (1.2)	
Perampanel	6 (5.4)	2 (1.2)	
Topiramate	20 (18)	11 (6.4)	
Lamotrigine	56 (50.5)	38 (22.1)	
*Therapeutic coma exposure*			
Duration of therapeutic coma, hours, median (IQR)	48 (24–84)		
Therapeutic coma duration, 24-h cycles, median (IQR)	2 (1–4)		
*Anesthetic agents used for therapeutic coma, n (%)*			
Propofol	95 (86.4)		
Midazolam	87 (79.1)		
Ketamine	40 (36.4)		
Inhalational anesthetics	10 (9.1)		
Barbiturates	43 (39.1)		

*ICU* indicates intensive care unit, *IQR* interquartile range, *LOS* length of stay

*Antiseizure medications (ASMs) ≥ 3 were considered a pragmatic proxy for refractory non-convulsive status epilepticus (NCSE)

^†^Among patients admitted to the intensive care unit

### In-hospital mortality and ICU length of stay

Overall, in-hospital mortality was 30.7% (87/283). Crude mortality was higher among patients treated with therapeutic coma than among those managed without coma (43.2% vs. 22.7%; *p* < 0.001). In IPTW-weighted analyses, therapeutic coma remained associated with higher in-hospital mortality. Weighted Poisson regression yielded a relative risk of 1.86 (95% CI 1.28–2.71; *p* = 0.001) and weighted logistic regression demonstrated an odds ratio of 2.52 (95% CI 1.44–4.42; *p* = 0.001). In IPTW-weighted linear regression of log-transformed ICU LOS, therapeutic coma was associated with a regression coefficient of 1.36 (95% CI 1.13–1.60; *p* < 0.001), corresponding to an approximately fourfold longer ICU stay. When additionally adjusting the IPTW-weighted mortality model for duration of SE, therapeutic coma remained independently associated with in-hospital mortality, although the association was attenuated (RR 1.59, 95% CI 1.08–2.34; *p* = 0.020). Longer SE duration was independently associated with higher mortality (RR per day 1.03, 95% CI 1.01–1.05; *p* = 0.001).

Cause-of-death categorization was available for 73 of 87 decedents (14 missing). Among decedents with available categorization, the distribution differed markedly by treatment group (*p* < 0.001). In the therapeutic coma group (*n* = 39), death occurred almost equally often following WLST (19/39, 48.7%) and under ongoing intensive care due to medical complications (20/39, 51.3%). In contrast, among patients managed without therapeutic coma (*n* = 34), the vast majority of deaths followed WLST (30/34, 88.2%), whereas death under ongoing intensive care due to complications was uncommon (4/34, 11.8%).

### Sensitivity analyses

Within (A) the subgroup of intubated patients (*n* = 145), therapeutic coma remained associated with higher odds of pneumonia (OR 4.77, 95% CI 1.72–13.19; *p* = 0.003), sepsis (OR 5.60, 95% CI 2.08–15.12; *p* = 0.001) and cardiac arrhythmias (OR 4.84, 95% CI 1.34–17.53; *p* = 0.016). Associations with acute renal failure (OR 3.61, 95% CI 0.98–13.34; *p* = 0.054) and renal replacement therapy (OR 6.87, 95% CI 0.86–54.76; *p* = 0.069) showed similar directions but did not reach statistical significance. Therapeutic coma remained associated with longer ICU length of stay (log-transformed ICU LOS: *β* = 0.342, 95% CI 0.059–0.626; *p* = 0.018). Associations with in-hospital mortality remained directionally consistent but no longer reached statistical significance (RR 1.71, 95% CI 0.92–3.20; *p* = 0.091; OR 2.29, 95% CI 0.95–5.55; *p* = 0.066).

In a further sensitivity analysis restricted to (B) patients treated with ≥ 3 ASMs (*n* = 169) as a proxy for refractory disease, therapeutic coma remained associated with higher in-hospital mortality (RR 2.01, 95% CI 1.14–3.52; *p* = 0.015; OR 2.75, 95% CI 1.28–5.90; *p* = 0.010) and prolonged ICU LOS (*β* = 0.97, 95% CI 0.66–1.28; *p* < 0.001). Therapeutic coma was also associated with increased odds of in-hospital medical complications, including pneumonia (OR 22.95, 95% CI 9.00–58.52; *p* < 0.001), sepsis (OR 9.78, 95% CI 3.30–28.95; *p* < 0.001), acute renal failure (OR 4.39, 95% CI 1.12–17.27; *p* = 0.034) and venous thromboembolism (OR 13.70, 95% CI 1.71–109.67; *p* = 0.014). For cardiac arrhythmia, renal replacement therapy and CPR, effect estimates could not be calculated due to complete separation, as events occurred exclusively in patients exposed to therapeutic coma.

After (C) exclusion of patients with hypoxic NCSE (*n* = 12), the associations between therapeutic coma and both primary and secondary endpoints remained robust.

Covariate balance for the propensity score models used in the sensitivity analyses is provided in Supplementary Tables S2–S4. Corresponding effect estimates from the sensitivity analyses are presented in Supplementary Figure S1.

## Discussion

In this large cohort of adult patients with NCSE, treatment with therapeutic coma was consistently associated with higher rates of medical complications, prolonged intensive care treatment and increased in-hospital mortality. These associations remained stable after adjustment for baseline differences using propensity score-based inverse probability of treatment weighting and across sensitivity analyses accounting for patients who required intubation and who had refractory disease. Notably, therapeutic coma was not associated with higher rates of sustained seizure termination before hospital discharge. These findings further highlight an important distinction between convulsive and non-convulsive forms of SE. While therapeutic coma is an established and often life-saving intervention in generalized convulsive SE, the balance of risks and benefits in NCSE appears less clear.

The most clinically relevant finding of this study is the significantly higher rate of medical complications. After adjustment, therapeutic coma was independently associated with increased risks of pneumonia, sepsis, cardiac arrhythmias, acute renal failure, need for renal replacement therapy, venous thromboembolism and CPR. The observed pattern of complications is biologically plausible in the context of deep sedation, mechanical ventilation and prolonged immobilization [20]. However, in a sensitivity analysis restricted to intubated patients, therapeutic coma remained independently associated with higher risks of pneumonia, sepsis and cardiac arrhythmias, as well as increased in-hospital mortality and longer ICU length of stay. Although effect sizes were attenuated compared with the primary analysis, the direction and consistency of associations suggest that the excess complication burden cannot be explained by mechanical ventilation alone, but rather reflects additional risks related to deep and prolonged anesthetic suppression. Similar complication profiles have been reported in prior observational studies of refractory and super-refractory generalized convulsive SE, in which infectious, cardiovascular and multiorgan complications were frequent among patients exposed to therapeutic coma [21, 22]. Importantly, the vast majority of studies evaluating therapeutic coma have focused on patients with generalized convulsive SE, whereas evidence specific to NCSE is extremely limited and largely confined to small observational case series or heterogeneous mixed cohorts in which NCSE-specific effects were not separately analyzed [5, 23]. Within the therapeutic coma subgroup, longer exposure to anesthetic treatment was associated with a dose-dependent increase in infectious complications. Each additional 24-h coma cycle was associated with a substantial increase in the odds of pneumonia, with a similar but statistically non-significant trend observed for sepsis. Although these dose–response analyses are exploratory and limited by sample size, they are consistent with prior reports linking prolonged mechanical ventilation and deep sedation to an incremental risk of ICU-acquired infections [24–26]. These findings underscore that treatment duration itself may represent a clinically relevant contributor to morbidity in NCSE.

Therapeutic coma was also associated with more than a twofold increase in the relative risk of death and an absolute mortality increase of approximately 24%. These effect sizes were robust across different modeling approaches, including weighted Poisson and logistic regression analyses. Although residual confounding cannot be excluded, particularly in the context of treatment escalation for more severe disease, the consistency of the association across adjusted and weighted models suggests that confounding by indication is unlikely to entirely account for the observed relationship. Prior observational work has similarly reported that greater treatment aggressiveness, including the use of therapeutic coma, is associated with worse outcomes in non-convulsive forms of SE, raising concern that iatrogenic harm may outweigh potential benefits in this population [27]. Therapeutic coma was further associated with a fourfold longer ICU stay. Prolonged ICU exposure itself carries well-established risks, including infectious and systemic complications and may represent an important pathway linking aggressive treatment escalation to adverse outcomes. Whether therapeutic coma directly contributes to prolonged critical illness or primarily reflects underlying disease severity cannot be resolved in this exploratory analysis.

Importantly, longer duration of SE itself was independently associated with higher in-hospital mortality and increased risks of infectious complications. This underscores the complex interplay between disease severity, seizure burden and treatment escalation. Prolonged seizure activity may contribute to systemic complications, while continuous anesthetic therapy may further amplify this risk.

The analysis of circumstances of death revealed a qualitative difference between treatment groups. In the therapeutic coma group, deaths occurred almost equally often under ongoing full intensive care due to medical complications as after withdrawal or limitation of life-sustaining treatment. In contrast, in patients managed without coma, most deaths followed explicit treatment limitation decisions, and death under ongoing maximal therapy was rare. Although retrospective classification of cause of death has inherent limitations, this pattern suggests that excess mortality in the coma group was not primarily driven by earlier or more frequent withdrawal of care, but rather by fatal complications occurring despite continued aggressive treatment.

Therapeutic coma was not associated with higher rates of sustained seizure termination before hospital discharge. In adjusted analyses, the likelihood of seizure resolution was lower in patients treated with therapeutic coma. This finding must be interpreted carefully, as therapeutic coma was preferentially used in patients with more refractory or severe disease. Importantly, refractory disease could only be approximated in this retrospective NCSE cohort. In contrast to generalized convulsive SE, refractoriness in NCSE cannot be judged clinically and relies on EEG monitoring, which is often intermittent or unavailable. We therefore used escalation to ≥ 3 antiseizure medications as a pragmatic proxy for refractory NCSE, recognizing that this approach may incompletely reflect true electrographic persistence.

Nonetheless, in this observational cohort we did not observe an association between therapeutic coma and improved short-term seizure termination. Whether this reflects limited treatment effectiveness, residual confounding by disease severity, or both cannot be determined from the present study. Treatment intensity further illustrates this complexity: patients receiving therapeutic coma required substantially more antiseizure medications, with most receiving three or more agents and a higher maximum number of concurrently administered drugs. Despite this greater treatment intensity, higher rates of short-term seizure termination were not observed in the therapeutic coma group. Given the observational nature of this study and the likelihood of residual confounding by disease severity, these findings should not be interpreted as evidence that treatment escalation is ineffective, but rather that a clear benefit of therapeutic coma on short-term seizure termination could not be demonstrated in this cohort.

Several limitations must be acknowledged. First, this study was retrospective and single-center in design. Although we applied propensity score-based inverse probability weighting and performed multiple sensitivity analyses to reduce measured confounding, residual confounding from unmeasured clinical factors cannot be excluded. The decision to initiate therapeutic coma was made at the discretion of the treating physicians and may have been influenced by unmeasured clinical factors such as perceived refractoriness, EEG pattern evolution or systemic instability. Second, although diagnoses were established in a specialized tertiary EEG center, NCSE classification relied on contemporaneous routine clinical EEG interpretations rather than centralized retrospective re-adjudication according to a single uniform framework; therefore, some diagnostic heterogeneity cannot be excluded. Analyses were based on available-case data without imputation. Variables related to therapeutic coma duration were missing in patients not treated with coma and were therefore analyzed exclusively within the coma subgroup. Given that all patients in the therapeutic coma group required intubation, it was not considered for adjustment in multivariable analyses. Third, long-term functional and cognitive outcomes were not available. In addition, treatment strategies evolved over the ten-year study period, which may have influenced both escalation thresholds and complication management. Despite these limitations, the present study provides detailed, NCSE-specific data on treatment intensity, complications and mortality in a relatively large cohort. The consistency of findings across multiple analytic approaches strengthens the internal coherence of the results. Ultimately, only prospective randomized studies can determine whether the observed associations reflect treatment effects or residual confounding. The ongoing FAST randomized controlled trial is expected to provide important evidence regarding the efficacy and safety of therapeutic coma in refractory NCSE [28].

In conclusion, in this retrospective cohort of patients with NCSE, therapeutic coma was associated with increased rates of medical complications, prolonged ICU treatment and higher in-hospital mortality, without an observed association with improved short-term seizure termination. While causal inference is not possible, our findings underscore the uncertainty regarding the benefit-risk balance of therapeutic coma in NCSE and highlight the need for prospective randomized studies to identify patients most likely to benefit from treatment escalation.

## Supplementary Information

Below is the link to the electronic supplementary material.

Supplementary file1 (DOCX 187 kb)

## Data Availability

All data generated or analyzed during this study are included in this published article.
