# Supplementary material for: Therapeutic coma in non-convulsive status epilepticus is associated with increased complications and mortality: a retrospective cohort study
Source: J Neurol. 2026 Sep 6;273(10):563. doi: 10.1007/s00415-026-14090-8 (PMC13546353; doi:10.1007/s00415-026-14090-8)
Supplement: Supplementary file 1 [file 415_2026_14090_MOESM1_ESM.docx]

| **Baseline characteristic** | **Before IPTW (absolute SMD)** | **After IPTW (absolute SMD)** |
| --- | --- | --- |
| Age | 0.471 | 0.080 |
| Female sex | 0.262 | 0.037 |
| NCSE with coma | 0.587 | 0.038 |
| History of epilepsy | 0.170 | 0.028 |
| Pre-admission ASM use | 0.260 | 0.038 |
| Etiology: Acute | 0.273 | 0.013 |
| Etiology: Remote | 0.105 | 0.005 |
| Etiology: Progressive | 0.288 | 0.003 |
| Etiology: Defined electroclinical syndrome | 0.075 | 0.003 |
| Etiology: Unknown | 0.030 | 0.021 |

**Supplementary Tables**

**Table S1.** Covariate balance before and after inverse probability of treatment weighting (IPTW) in the primary propensity score analysis.

Abbreviations: ASM, antiseizure medication; IPTW, inverse probability of treatment weighting; NCSE, non-convulsive status epilepticus; SMD, standardized mean difference.

**Table S2.** Covariate balance before and after inverse probability of treatment weighting (IPTW) in the sensitivity analysis restricted to mechanically ventilated patients

| **Baseline characteristic** | **Before IPTW (absolute SMD)** | **After IPTW (absolute SMD)** |
| --- | --- | --- |
| Age | 0.400 | 0.195 |
| Female sex | 0.064 | 0.072 |
| NCSE with coma | 0.181 | 0.059 |
| History of epilepsy | 0.095 | 0.114 |
| Pre-admission ASM use | 0.006 | 0.035 |
| Etiology: Acute | 0.095 | 0.021 |
| Etiology: Remote | 0.113 | 0.036 |
| Etiology: Progressive | 0.375 | 0.049 |
| Etiology: Defined electroclinical syndrome | 0.236 | 0.144 |
| Etiology: Unknown | 0.294 | 0.097 |

Abbreviations: ASM, antiseizure medication; IPTW, inverse probability of treatment weighting; NCSE, non-convulsive status epilepticus; SMD, standardized mean difference.

**Table S3.** Covariate balance before and after inverse probability of treatment weighting (IPTW) in the sensitivity analysis restricted to patients treated with ≥3 antiseizure medications

| **Baseline characteristic** | **Before IPTW (absolute SMD)** | **After IPTW (absolute SMD)** |
| --- | --- | --- |
| Age | 0.326 | 0.033 |
| Female sex | 0.339 | 0.000 |
| NCSE with coma | 0.452 | 0.008 |
| History of epilepsy | 0.073 | 0.008 |
| Pre-admission ASM use | 0.148 | 0.022 |
| Etiology: Acute | 0.164 | 0.017 |
| Etiology: Remote | 0.018 | 0.030 |
| Etiology: Progressive | 0.302 | 0.007 |
| Etiology: Defined electroclinical syndrome | 0.081 | 0.007 |
| Etiology: Unknown | 0.008 | 0.012 |

Abbreviations: ASM, antiseizure medication; IPTW, inverse probability of treatment weighting; NCSE, non-convulsive status epilepticus; SMD, standardized mean difference.

**Table S4.** Covariate balance before and after inverse probability of treatment weighting (IPTW) after exclusion of patients with hypoxic NCSE

| **Baseline characteristic** | **Before IPTW (absolute SMD)** | **After IPTW (absolute SMD)** |
| --- | --- | --- |
| Age | 0.435 | 0.078 |
| Female sex | 0.268 | 0.050 |
| NCSE with coma | 0.577 | 0.032 |
| History of epilepsy | 0.137 | 0.030 |
| Pre-admission ASM use | 0.234 | 0.044 |
| Etiology: Acute | 0.213 | 0.009 |
| Etiology: Remote | 0.059 | 0.004 |
| Etiology: Progressive | 0.274 | 0.001 |
| Etiology: Defined electroclinical syndrome | 0.124 | 0.000 |
| Etiology: Unknown | 0.065 | 0.019 |

Abbreviations: ASM, antiseizure medication; IPTW, inverse probability of treatment weighting; NCSE, non-convulsive status epilepticus; SMD, standardized mean difference.

**Supplementary Figures**

**Figure S1.** Association between therapeutic coma and in-hospital medical complications among mechanically ventilated patients.

Squares represent odds ratios and horizontal lines indicate 95% confidence intervals derived from IPTW-weighted logistic regression models. Odds ratios for cardiopulmonary resuscitation and venous thromboembolism could not be estimated because these events occurred exclusively among patients treated with therapeutic coma (complete separation).

**Figure S2.** Association between therapeutic coma and in-hospital medical complications among patients treated with ≥3 antiseizure medications.

Squares represent odds ratios and horizontal lines indicate 95% confidence intervals derived from IPTW-weighted logistic regression models. Odds ratios for cardiac arrhythmias, renal replacement therapy and cardiopulmonary resuscitation could not be estimated because these events occurred exclusively among patients treated with therapeutic coma (complete separation).

**Figure S3.** Association between therapeutic coma and in-hospital medical complications after exclusion of patients with hypoxic non-convulsive status epilepticus.

Squares represent odds ratios and horizontal lines indicate 95% confidence intervals derived from IPTW-weighted logistic regression models.
